# Presepsin: An Emerging Biomarker in the Management of Cardiometabolic Disorders

**DOI:** 10.3390/jpm15040125

**Published:** 2025-03-25

**Authors:** Dimitrios Kouroupis, Ioanna Zografou, Panagiotis Doukelis, Dimitrios Patoulias, Djordje S. Popovic, Paschalis Karakasis, Athina Pyrpasopoulou, Konstantinos Stavropoulos, Christodoulos Papadopoulos, Olga Giouleme, Kalliopi Kotsa, Michael Doumas, Theocharis Koufakis

**Affiliations:** 1 Second Propedeutic Department of Internal Medicine, Hippokration General Hospital, Aristotle University of Thessaloniki, 54642 Thessaloniki, Greece; dimcour841@gmail.com (D.K.); ioannazo@yahoo.gr (I.Z.); pitdukel@yahoo.gr (P.D.); dipatoulias@gmail.com (D.P.); a.pyrpasopoulou@doctors.org.uk (A.P.); konvstavropoulos@hotmail.com (K.S.); doumasm@auth.gr (M.D.); 2 Clinic for Endocrinology, Diabetes and Metabolic Disorders, Clinical Centre of Vojvodina, Medical Faculty, University of Novi Sad, 21000 Novi Sad, Serbia; djordje.popovic@mf.uns.ac.rs; 3 Second Department of Cardiology, Aristotle University of Thessaloniki, Hippokration General Hospital, 54642 Thessaloniki, Greece; pakar15@hotmail.com; 4 Third Department of Cardiology, Hippokration General Hospital, Aristotle University of Thessaloniki, 54642 Thessaloniki, Greece; chpapado@auth.gr; 5 Second Propedeutic Department of Internal Medicine, Hippokration General Hospital, Gastroenterology and Hepatology Division, Medical School, Aristotle University of Thessaloniki, 54642 Thessaloniki, Greece; giouleme@auth.gr; 6 Division of Endocrinology and Metabolism and Diabetes Centre, First Department of Internal Medicine, Medical School, Aristotle University of Thessaloniki, AHEPA University Hospital, 54636 Thessaloniki, Greece; kkalli@auth.gr

**Keywords:** presepsin, diabetes, inflammation, biomarker, innate immune system, cardiovascular disease

## Abstract

**Background/Objectives:** Systemic and tissue inflammation play a crucial role in the pathophysiology of cardiometabolic disorders. Presepsin is a newly discovered marker of acute phase inflammation that is produced by monocytes or macrophages in response to bacterial infection and is a soluble fraction of the lipopolysaccharide (LPS) receptor. LPS is an endotoxin that, through the breakdown of the intestinal barrier, penetrates the systemic circulation and is an important bacterial mediator in the pathogenesis of sepsis and septic shock. **Methods:** A narrative review of the existing literature. **Results:** A growing body of evidence demonstrates that intestinal dysbiosis is involved in the pathogenesis of diabetes mellitus (DM) and cardiovascular (CV) disease, leading to increased circulating LPS concentrations in people with cardiometabolic disorders, even in the absence of infection. These data provide the theoretical background for a link between presepsin, DM, and CV pathology. Preliminary studies suggest that presepsin levels are downregulated in patients with well-controlled type 2 DM and correlate with continuous glucose monitoring metrics in infection-free individuals with type 1 DM. However, prospective data on the association between presepsin and the risk of diabetic complications are currently lacking. Presepsin has also been found to be elevated in infection-free individuals with myocardial infarction, heart failure, and myocarditis compared to controls and has been shown to correlate with mortality risk in subjects at high CV risk. **Conclusions:** The clinical utility of presepsin in the monitoring of patients with cardiometabolic disorders warrants further investigation by future studies.

## 1. Introduction

Cardiovascular (CV) disease poses an increasing challenge to healthcare systems globally [1]. Cardiometabolic disease is a systemic disorder characterized by pathophysiological interactions between metabolic risk factors and the CV system, leading to multiorgan dysfunction and an increased incidence of adverse clinical outcomes [2]. Recent developments in the management of CV conditions are primarily attributed to innovative imaging technologies that enhance diagnosis and risk assessment. Nevertheless, there is a widespread acknowledgment of the necessity to identify new biomarkers that can facilitate early detection, accurate diagnosis, and prognosis, as well as tailored treatment approaches [3].

Diabetes mellitus (DM) is one of the most common metabolic disorders globally and a major factor in the development of CV disease. By 2045, approximately 12% of the adult population around the world is expected to live with DM, increasing from 10.5% in 2021 [4]. Poor glycemic control is associated with life-threatening complications, with DM being today the leading cause of end-stage renal disease, non-traumatic amputations, blindness, and a major driver of CV morbidity and mortality [5]. These data highlight the need for close monitoring of glycemia in people with DM in order to avoid long-term complications of the disease that have a negative impact on quality of life and pose an important challenge to healthcare systems [6].

For many years, measuring glycated hemoglobin (HbA1c) has been the gold standard method to evaluate the quality of glycemic control in people with DM. There is no doubt that HbA1c has numerous advantages, being a widely accessible, relatively inexpensive, and practical method of assessing glycemia. Additionally, physicians are familiar with the interpretation of the test and its clinical implications. Most importantly, landmark prospective studies have shown that HbA1c levels correlate well with the risk of microvascular diabetic complications (retinopathy, nephropathy, and neuropathy) in populations with type 1 DM (T1DM) [7] and type 2 DM (T2DM) [8]. However, HbA1c is not free of limitations, since its accuracy is affected by common medical conditions, including anemia, recent blood transfusions, and vitamin C intake [9]. Furthermore, it cannot reflect glycemic variability, which is increasingly recognized as an important contributing factor to the development of diabetic complications [10,11].

The limitations of HbA1c, along with the introduction of technology in the management of DM, have led to the adoption of continuous glucose monitoring (CGM) as an alternative method of evaluating glycemic control, particularly in patients with T1DM or those with T2DM using complex insulin regimens. The use of CGM has been associated with a lower risk of hypoglycemia and glycemic variability, improved quality of life, and possibly better glycemic control [12]. There is evidence that time in range (TIR), that is, the amount of time people with diabetes spend with their blood glucose levels in a recommended target range, is correlated with the risk of complications [13]; however, robust data from large prospective trials are still lacking. On the other hand, the increased cost to patients and healthcare systems, the need for continuous education to use these devices effectively, the development of alarm fatigue, and the reluctance of some people to wear devices in visible parts of their body are some of the drawbacks of CGM [14].

For the reasons mentioned above, the pursuit of the ideal marker for the monitoring of patients with cardiometabolic diseases is ongoing. The purpose of the present article is to review preliminary evidence on the role of presepsin, a marker previously exclusively associated with sepsis, in the pathophysiology, prognosis, and management of cardiometabolic disorders.

## 2. The Role of Inflammation in Diabetes and Cardiovascular Disease

It is well-established that inflammation is a key mechanism in the development of hyperglycemia and its complications. A consequence of excess energy intake in people with obesity and T2DM is the inability of fat storage to handle the superfluous amount of energy, leading to ectopic fat accumulation in the liver, pancreas, muscles, perivascular tissue, and pericardium [15]. Endoplasmic reticulum stress of hypertrophic adipocytes accelerates their apoptosis, transforming dying adipocytes into antigens that, through the production of adipokines and chemoattractants, attract activated immune cells, such as macrophages, in adipose tissue [16]. The pro-inflammatory cascade results in the upregulation of cytokines, such as tumor necrosis factor a (TNF-a), which are known to promote insulin resistance and pancreatic cell death [17].

In addition to the traditional concept of adipose tissue dysfunction, the role that gut dysbiosis plays in the generation and maintenance of inflammation in DM has gained increasing interest in the past decade. This is a bidirectional relationship, with gut imbalance aggravating DM and vice versa. Alterations in the intestinal microbiota observed in people with T2DM and obesity are believed to result in the breakdown of the intestinal barrier, which, in turn, allows the penetration of gut-origin antigens into the systemic circulation, activating immune cells and fueling the vicious cycle of inflammation [18]. One of the most potent activators of the immune system is Gram-negative bacteria lipopolysaccharide (LPS), which is considered an important bacterial mediator in the pathogenesis of sepsis and septic shock [19]. Large adipocytes with increased metabolic activity are known to absorb LPS-rich lipoproteins, while macrophages in adipose tissue internalize LPS-lipoproteins [20]. Increased delivery of LPS to hypertrophic adipose tissue triggers macrophage polarization from the M2 to M1 phenotype, which is characterized by the production of high levels of pro-inflammatory cytokines [21]. Preliminary evidence suggests a positive correlation between circulating LPS levels in infection-free people with DM, a phenomenon known as metabolic endotoxemia, and the risk of diabetic complications [22]. In a cross-sectional study that included 482 men with metabolic syndrome (MetS), Awoyemi et al. [23] found a positive association between waist circumference (WC) values and LPS binding protein (LBP) levels, suggesting a possible role for the innate immune system in the genesis of metabolic dysregulation. In the same study, a significant trend toward an increased prevalence of MetS with ascending quartiles of LBP was observed. In patients with obesity who underwent metabolic surgery, plasma levels of LPS were elevated compared to lean controls and decreased after the procedure, while a close correlation was observed between LPS, HbA1c, and intra-abdominal fat volume [24]. A systematic review by Moludi et al. [25] suggested that prebiotics and probiotics can potentially improve CV health by suppressing endotoxin, thus alleviating metabolic endotoxemia and related systemic inflammation; however, the authors emphasize that the existing evidence is still weak to support definitive conclusions.

C-reactive protein (CRP) is one of the most well-investigated markers of inflammation in cardiometabolic disorders. A meta-analysis of 22 cohorts with a total of 40,735 participants found that elevated CRP levels are associated with an increased risk of T2DM (relative risk 1.26) [26]. In the EPIC-Norfolk cohort, serum CRP was associated with the risk of developing DM after adjusting for multiple confounders, including age, sex, body mass index (BMI), smoking, and physical activity [27]. However, the association attenuated after the authors controlled for the effects of central adiposity, markers of liver dysfunction, and adiponectin levels, challenging the notion that CRP could be an independent risk factor for T2DM. It has been debated whether an increase in CRP is causally related to CV disease or simply represents an epiphenomenon [28]. On the other hand, robust evidence indicates that CRP is an independent risk factor for CV disease and mortality. A sub-analysis of the CANTOS trial, in which canakinumab was administered to patients with a history of myocardial infarction, showed that in participants who achieved CRP concentrations lower than 2 mg/L, both CV and all-cause mortality decreased by 31% [29].

Procalcitonin (PCT), another well-known sepsis biomarker, has been positively associated with insulin resistance, BMI, WC, and components of metabolic syndrome [30]. In a cross-sectional study from Sub-Saharan Africa, serum PCT was shown to have a higher sensitivity and specificity than high-sensitivity CRP (hsCRP) in detecting people at high CV risk [31]. Using data from the Prevention of Renal and Vascular End-stage Disease (PREVEND) study in the Netherlands, Abbasi et al. [32] showed that PCT is an independent predictor of incident T2DM in the general population, also having a stronger association with the risk of DM than hsCRP after accounting for adiposity.

In patients with acute heart failure (HF) and without evidence of infection, PCT was positively and independently associated with the risk of death and both HF-related and all-cause recurrent rehospitalizations [33]. It is believed that the link behind the upregulation of PCT levels in HF is increased endotoxin release from the congested intestine as a consequence of impaired blood supply to the intestinal tract. In support of this perspective, Mollar et al. [34] have shown that in subjects with acute HF, kidney function and surrogates of venous congestion are the main determinants of PCT concentrations. Another link between CV disorders and acute inflammation lies in the fact that the reduction in oxygen availability to the myocardium and subsequent myocyte necrosis are predominantly regulated by monocytes and macrophages [35]. It appears that neutrophils are instrumental in the processes of myocardial reperfusion injury and constructive remodeling of myocardial tissue after cardiac ischemia [36].

The role of TNF-a in the pathophysiology of CV disorders is well investigated. Increased TNF-a is believed to downregulate the activity of the eNOS gene, resulting in reduced nitric oxide production and, subsequently, in the inhibition of endothelium-dependent vasodilation [37]. Inhibition of TNF-a by administration of the monoclonal antibody adalimumab was shown to significantly improve markers of endothelial function in patients with psoriasis [38]. Proinflammatory cytokines, including interleukins (IL)-1 and 6, increase sympathetic tone by enhancing endothelin-1 release, thus contributing to the development of arterial stiffness [39]. Following acute ST elevation myocardial infarction (STEMI), IL-6 has been shown to be an important driver of left ventricular geometry alterations that result in the development of HF [40]. IL-18 levels have been shown to predict the risk of atherosclerotic plaque rupture [41] while circulating IL-6 concentrations were positively associated with the risk of CV and all-cause mortality in elderly individuals [42].

The neutrophil-to-lymphocyte ratio (NLR) is a surrogate marker of systemic inflammation, reflecting the imbalance between the pro-inflammatory and fibrinolytic pathways [43]. Elevated NLR values have been associated with increased vulnerability of carotid plaque assessed by ultrasound and a higher risk of ischemic stroke among Chinese patients [44]. Oxidative damage induced by the release of reactive oxygen species by circulating neutrophils is believed to be an important underlying mechanism [45]. Lin et al. [46] have shown that NLR is an independent predictor of CV death in individuals with STEMI and is also positively correlated with the magnitude of ventricular dysfunction. Finally, increased fibrinogen levels have been shown to be associated with larger infarct size in STEMI patients [47].

However, the link between inflammation and CV disease is complex, and many aspects remain poorly understood. To support this perspective, some studies have produced negative results on a definitive association between inflammation and CV disorders. Defteros et al. [48] did not show a clear benefit from colchicine treatment, an anti-inflammatory agent, in improving functional status, measured by the New York Heart Association scale, or mortality rates in patients with stable chronic HF. This is notable despite colchicine’s proven effectiveness in preventing the recurrence of atrial fibrillation [49]. Therefore, further research is needed on the role of inflammatory pathways in the development of CV disease to improve our understanding of the biomarkers involved and their relationship with systemic inflammatory disorders [39].

## 3. Presepsin as a Biomarker of Bacterial Infections

Presepsin is identified as a subtype of the soluble form of CD14 (sCD14-ST), specifically comprising its 13 kDa N-terminal fragment, which is instrumental in the activation of the innate immune system. Recent research conducted over the last decade has demonstrated that levels of presepsin rise in response to bacterial infections and subsequently decline following recovery or effective treatment [50], thereby positioning presepsin as a promising biomarker for the early identification of infections. Infections initiate the host’s immune response, which is generally divided into innate and adaptive systems. Both systems necessitate the recognition of pathogens; however, the innate immune system employs pre-existing receptors on the surfaces of immune effector cells to detect a wide array of antigens present in most microbial pathogens. CD14, a 55 kDa transmembrane glycoprotein, serves as a coreceptor on monocytes and macrophages and is part of the Toll-like receptor family, essential for recognizing various ligands from both Gram-positive and Gram-negative bacteria. The recognition of LPS necessitates the participation of LBP, which facilitates the presentation of LPS to CD14 [51]. The subsequent formation of the CD14-LPS-LBP complex activates intracellular signaling pathways that promote the expression of genes associated with the immune response, including the production of cytokines by effector cells. CD14 is present in two forms: a membrane-bound variant (mCD14) found in monocytes and macrophages, and a soluble variant (sCD14) found in plasma, which is cleaved by cathepsin D into the 13 kDa fragment (sCD14-ST) that circulates within the bloodstream [52].

Presepsin can normally be detected in the plasma of infection-free subjects, however, at low concentrations. A study that included healthy individuals identified the reference limits between 55 and 184 pg/mL, with the 90% confidence interval ranging from 45 to 58 for the lower limit and from 161 to 214 for the upper limit. No significant differences between men and women were observed [53]. After bacterial infection, presepsin increases in a few hours, and its levels positively correlate with the intensity of the immune response [54]. According to Lee et al. [55], the optimal cut-off value for the diagnosis of sepsis is 582 pg/mL, while concentrations higher than 1285 pg/mL are suggestive of septic shock. In the same study, levels above 821 pg/mL were associated with a higher mortality rate compared to lower values. Although initially believed to be exclusively a marker of Gram-negative sepsis, emerging data suggest that presepsin levels can also increase in response to Gram-positive, fungal, and parasitic infections [56].

Due to its low molecular weight, presepsin is filtered by the glomeruli, where it is then reabsorbed and proteolyzed in the proximal tubule. In healthy individuals, small amounts of presepsin produced in the absence of infection are removed from the circulation primarily by glomerular filtration in the kidneys [57]. Therefore, it would be anticipated that patients with renal failure [characterized by a decrease in the glomerular filtration rate (GFR)] would have higher concentrations of presepsin. This may be particularly pronounced in dialysis-dependent individuals with end-stage renal disease, as presepsin is not filtered during dialysis. In critically ill septic patients, a rapid increase in presepsin levels may be indicative of acute renal failure, which is a serious but common complication of sepsis. Subjects with stage 3, 4, and 5 renal failure had presepsin values significantly higher than those of the control group with normal renal function [58]. In addition to kidney function, presepsin levels may also be affected by age. In a study that recruited 144 consecutive patients who presented to the emergency department for a condition other than infection, age above 70 years was an independent predictor of elevated presepsin values [59].

Compared to CRP, IL-6, and PCT, presepsin was the only biomarker that remained elevated after the start of antibiotic treatment in the group presenting with the most serious infection and retained correlation with increased mortality risk until the end of the follow-up period [60]. A recent meta-analysis that included four studies and 308 children and adolescents found that presepsin has a higher sensitivity and diagnostic accuracy but lower specificity compared to CRP or PCT in detecting sepsis [61]. A recently published retrospective study that included patients with diabetic foot ulcers suggested that a cut-off value of 675 ng/mL can accurately predict serious infections that need amputation [62]. In patients undergoing total hip arthroplasty, a delayed decrease in presepsin concentrations more than 96 h after the procedure was associated with increased infection risk [63]. Finally, Botnariu et al. [64] studied 60 patients with DM and sepsis and found that presepsin is correlated with mortality rate, Simplified Acute Physiology Score II (a severity of disease classification system), serum fibrinogen values and markers of renal function (blood urea and creatinine). These findings indicate presepsin as a valuable biomarker for the early and accurate detection of sepsis in people with DM.

## 4. Presepsin in Cardiometabolic Diseases

**Figure 1:**
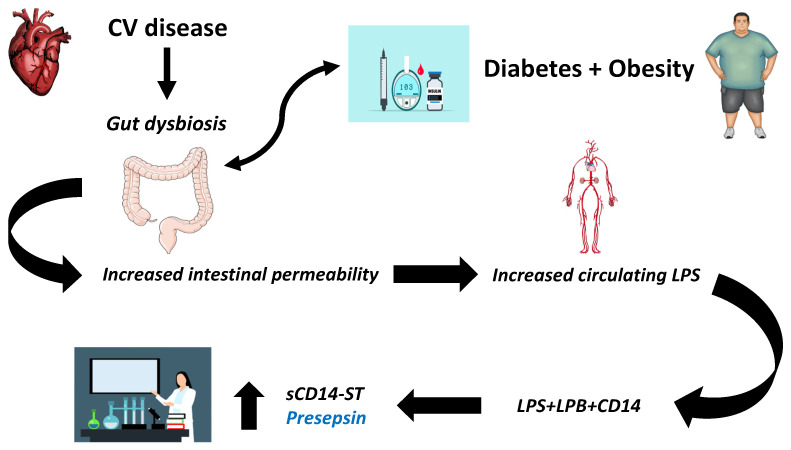
The pathophysiological rationale behind the use of presepsin as a biomarker in cardiometabolic disorders in the absence of infection. Abbreviations: CV: cardiovascular; LPS: lipopolysaccharide; LBP: lipopolysaccharide binding protein; sCD14-ST: soluble CD14 subtype.

The rationale behind the evaluation of presepsin levels in patients with DM, obesity, and other cardiometabolic disorders in the absence of infection lies in the phenomenon of metabolic endotoxemia. It is reasonable to assume that the increase in LPS levels as a consequence of intestinal dysbiosis (and the associated deterioration of the inflammatory state) should be accompanied by a respective upregulation of its receptor, which is presepsin (Figure 1). Although only a few studies have investigated this concept so far, preliminary results are encouraging.

In a recently published work, presepsin was measured in infection-free subjects with DM [65]. In this exploratory study, patients with well-controlled (*n* = 19) and poorly controlled T2DM (*n* = 23), individuals with well-controlled (*n* = 10) and uncontrolled (*n* = 10) T1DM, as well as normoglycemic controls (*n* = 13) were included. Exclusion criteria were age > 70 years, renal dysfunction (defined as GFR values < 60 mL/min/1.73 m^2^), presence of acute infections (in the last 3 months) or chronic infections, and a history of any inflammatory condition, including malignancies, autoimmune disorders, and recent surgery. Based on their HbA1c levels, participants with DM were classified into well (<7%) and poorly (≥7%) controlled groups and presepsin was determined with the ELISA method.

It was found that presepsin concentrations were associated with the duration of DM in multiple linear regression analysis (*p* = 0.008). Furthermore, presepsin levels were significantly lower in the well-controlled T2DM group compared to the adequately controlled T1DM group [1.34 (2.02) vs. 2.22 (4.20) ng/mL, *p* = 0.01]. This difference retained statistical significance after adjustment for various confounders, including BMI and WC. As expected, participants with T1DM were exclusively treated with insulin, in contrast to those with T2DM who were on various combinations of antidiabetic agents, including sodium-glucose cotransporter 2 inhibitors (SGLT2i) (47.3%), glucagon-like receptor agonists (GLP-1 RAs) (63.1%), metformin (84.2%), dipeptidyl peptidase 4 inhibitors (31.5%), and pioglitazone (5.2%). Furthermore, more patients with T2DM were on therapy with statins and antihypertensives compared to those with T1DM (78.8 vs. 7.69% and 89.4 vs. 15.3%, respectively, *p* < 0.001 in both cases). These findings are probably suggestive of the anti-inflammatory actions of the drugs used in the treatment of T2DM. A systematic review found that diabetes drugs reduce LPS levels, with the strongest effects exerted by thiazolidinediones and the weakest by insulin [66]. With respect to SGLT2i and GLP-1 RAs, particularly, there is evidence that they ameliorate systemic inflammation, a mechanism that is believed to mediate their impressive cardioprotective effects [67,68]. Although insulin also exerts anti-inflammatory actions, these are short-term and can be counteracted in the long term by its anabolic effects and the negative impact on body weight that fuels the vicious cycle of inflammation [69]. Statins, which are widely used to achieve cholesterol targets in the vast majority of patients with T2DM, are also known to mitigate inflammation, and this is believed to contribute to the cardioprotection offered by the class [70]. An alternative explanation for the higher concentrations of presepsin in people with T1DM compared to patients with T2DM is the overactivation of the immune system, particularly of cytotoxic T cells, which is the main mechanism of beta-cell damage and destruction in the autoimmune form of the disease. A recently published study from our group [71] showed that obesity in the absence of T2DM and infection is associated with elevated presepsin levels after adjustment for several confounders, such as age, sex, smoking status, and vitamin D levels. Interestingly, presepsin was found to be reduced in the overweight state, a finding that could be attributed to a counterregulatory mechanism aiming to protect from the increase in circulating LPS levels.

A different study [72] included 20 infection-free patients with T1DM (mean age 35.5 years) treated with insulin pumps (*n* = 6) or multiple daily injections (*n* = 14). Among the study participants, 12 used CGM. The patients were classified into well- and poorly controlled groups according to their recent HbA1c levels and from the last 14-day CGM recordings the following metrics were extracted: time in range (TIR; 70–180 mg/dL), time above range (TAR), time below range (TBR), glucose management indicator (GMI), coefficient of variation and average glucose values. Presepsin levels were similar between the well-controlled and poorly controlled groups [2.22 (4.20) vs. 2.48 (4.04) ng/mL, *p* = 0.43]. A trend toward a positive correlation was observed between presepsin values and GMI and a negative correlation between presepsin and TIR. Furthermore, a positive correlation was found with the Glycemia Risk Index (GRI), a composite metric of glycemia quality based on CGM traces [73]. GRI is based on weighted combinations of TBR (the hypoglycemia component) and TAR (the hyperglycemia component). Therefore, these preliminary data support the notion that presepsin could be a useful biomarker for monitoring glycemic control since its levels appear to change in response to high and low plasma glucose concentrations [72]. Given that CGM is free of some of the limitations of HbA1c in monitoring glycemia, particularly with respect to the reflection of glycemic variability, the relationship between presepsin and CGM metrics deserves further evaluation in larger studies.

Biyik et al. [74] investigated the association between presepsin levels and blood pressure control in 48 patients with adequately controlled hypertension (HT) and 48 normotensive controls. Among study participants, 22% in the HT group and 19.6% in the control group had T2DM. Regarding the antihypertensive treatment used by the study sample, 66.6% were on therapy with angiotensin-converting enzyme inhibitors (ACEi) or angiotensin receptor blockers (ARB), 29.1% with beta-blockers (BB), 20.8% with calcium channel blockers (CCB) and 37.5% with diuretics. Presepsin was found to be lower in patients with well-controlled HT compared to controls (144.98 ± 75.98 vs. 176.67 ± 48.12 pg/mL, *p* = 0.011), while its levels were positively correlated with hsCRP in both study groups. Similarly to what was observed in T2DM, it can be speculated that these findings are related to the anti-inflammatory effects of antihypertensive drugs. Previous studies have shown that ACEi, ARB, CCB, and BB have the potential to alleviate systemic and vascular inflammation [75,76]. In a retrospective analysis, Qi et al. [77] showed that presepsin levels were significantly higher in out-of-hospital cardiac arrest patients (22% had DM) compared to healthy controls. Furthermore, low presepsin concentrations were an independent prognostic factor for survival and favorable neurological outcomes in the patient group and were correlated with human leucocyte antigen-DR expression in monocytes. These interesting data indicate that cardiac arrest is characterized by an impaired immunological response that is very similar to sepsis and underlines the common pathogenetic pathways between infections and CV disease, where inflammation is the interface between the two entities.

In a study that included 222 patients with coronary artery disease (CAD) who underwent major noncardiac surgery, increased presepsin levels before the procedure were an independent predictor of major adverse CV and cerebrovascular events (CV death, myocardial infarction, myocardial ischemia, and stroke) during the 30-day postoperative period [78]. Presepsin was also shown to be an independent predictor of perioperative CV events after adjustment for confounders. In another study by the same group that enrolled 40 patients at high CV risk subjected to noncardiac surgery, a presepsin cut-off greater than 184 pg/mL was shown superior to high-sensitive cardiac troponin T in complementing the N-terminal prohormone of brain natriuretic peptide (NT-proBNP) for the prediction of the risk of perioperative CV events [79]. A study [80] that included 50 patients with decompensated HF and 51 controls showed that presepsin levels were significantly higher in the HF group than in the control group, while a cutoff of 442 pg/mL had a 76% sensitivity, a 62.7% specificity, a positive predictive value of 66.7% and a negative predictive value of 72.7% for the detection of HF. However, the diagnostic accuracy of presepsin was not superior to that of NT-proBNP. In patients hospitalized with worsening HF, Nishimura et al. [81] showed that presepsin levels correlate significantly with eGFR, NT-proBNP, and hsCRP concentrations, also being an independent predictor of 6-month mortality.

In a large prospective study that included 1636 patients (46% with CAD and 36% with decompensated HF) treated in cardiac intensive care units, presepsin levels were strongly associated with the risk of mortality (*p* < 0.00001) over a mean follow-up period of 44.6 months [82]. The addition of presepsin to a model that also included hsCRP and BNP seemed to further improve its predictive value. A recent study from Turkey evaluated presepsin in 68 patients with acute myocarditis and 70 controls [83]. The authors found that presepsin levels were significantly higher in the patient than in the control group, while in a regression analysis, presepsin was shown to be an independent predictor of myocarditis. Another small study that enrolled 48 patients with acute STEMI and 50 healthy controls found significantly higher presepsin values in the STEMI group compared to the control group [84]. The presepsin threshold of 447 pg/mL had a sensitivity of 87.5%, a specificity of 44%, a positive predictive value of 60%, and a negative predictive value of 78.5% for the detection of STEMI. Finally, a prospective study evaluated presepsin and BNP in 239 outpatients (41% had DM) with chronic kidney disease that did not require dialysis [85]. Presepsin values in the highest tertile (>480 pg/mL) were associated with a higher risk of CV mortality and morbidity during a mean follow-up period of 24.1 months. Collectively, these findings support a wider role for presepsin in the treatment of cardiometabolic disorders, such as DM, HT, HF, and CAD, which very frequently coexist and have an additively negative impact on CV risk and mortality [86]. Table 1 provides an overview of the studies that investigated presepsin in infection-free subjects with cardiometabolic disorders.

## 5. Conclusions and Future Perspectives

The ideal biomarker should have specific characteristics. It must be visible early before the histopathological changes become apparent. It should be sensitive, specific for a certain disease or disorder, accessible in peripheral tissues (preferably measurable in blood), and associated with a known pathophysiological mechanism [87]. Furthermore, it must be practical, useful for the prediction of disease-related outcomes and follow-up of patients, cost-effective, easy to interpret, and correlate with the risk of complications in order to find its way into clinical practice. However, it should be kept in mind that the perfect biomarker in the management of cardiometabolic disorders probably does not exist; therefore, research should focus on the identification of markers with high specificity and sensitivity, together with low cross-reactivity. Furthermore, the reliability of biomarkers should always be confirmed by alternative methods. In this context, the correlation of presepsin values with CGM metrics reinforces the notion that presepsin might be useful in monitoring glycemia.

Currently, and considering the paucity of relevant data, it is dubious whether presepsin gathers the desirable features to become a useful biomarker in the management of DM and CV disease. It reflects a key pathophysiological disturbance, intestinal dysbiosis, that is common throughout the cardiometabolic spectrum and is a good predictor of adverse outcomes in patients at high CV risk. This gives presepsin the potential to become a uniform marker in the detection and treatment of DM [65,72], obesity [65,71], HT [74], and CV disorders [77,78,79,80,81,82,83,84,85]. Preliminary data show that it correlates well with the quality of glycemic control, is associated with the duration of DM, is affected by both hyperglycemic and hypoglycemic components, and is influenced by antidiabetic and antihypertensive medications, which could be useful for monitoring the effectiveness of therapeutic strategies in cardiometabolic diseases.

On the other hand, there are several issues that need to be resolved and clarified before the clinical use of presepsin as a biomarker in cardiometabolic diseases. Commercial kits for presepsin measurement are still costly and not widely accessible, restricting their use mainly for research purposes. Although presepsin can be blamed for its low specificity given that it increases in heterogeneous disorders, preliminary evidence suggests that diagnostic cut-offs are different, with lower values associated with metabolic diseases and higher concentrations with infections. It is possible that different concentrations can also discriminate DM from obesity or CV disease; however, more research is needed on this issue. The available studies are small, have a cross-sectional or retrospective design, and therefore cannot establish causal associations. Most importantly, unlike the current gold standard biomarker for monitoring glycemic control, which is HbA1c, data on the association between presepsin and the risk of complications are lacking.

It should be noted that currently there are no studies that evaluate presepsin comparatively with traditional and well-established biomarkers, including CRP, PCT, and HbA1c, in infection-free patients with DM. Regarding the comparison with HbA1c, it could be said that the main advantage of presepsin is its correlation with glycemic variability, which is considered an important driver of diabetic complications. On the contrary, HbA1c cannot reveal large fluctuations in blood glucose levels, mainly reflecting its mean values over the past 3 months. Regarding CRP and PCT, it seems that their relationship with DM can be confounded by the volume and distribution of adipose tissue [27], underscoring the need to evaluate biomarkers that link inflammation and DM through alternative pathways. With respect to the field of CV medicine, the limitations of existing biomarkers are characterized by a lack of specificity, as they may be elevated in various diseases, complicating the identification of the precise origin of a patient’s symptoms. Furthermore, certain biomarkers may lack the sensitivity required to identify diseases in their initial stages, leading to delays in diagnosis and treatment, which is particularly critical in the context of CV disease, where timely therapy is often life-saving [88]. Biomarker levels can exhibit considerable variability among individuals, attributed to both genetic and epigenetic factors, which presents challenges in establishing universal cut-off values for diagnosis and risk evaluation. Considering the paucity of the available evidence, it is still uncertain whether presepsin is free of the above limitations when used as a biomarker in CV disorders. However, it is important to recognize that the various biomarkers used in the management of a disease are not necessarily competitive but can be conjointly evaluated as they can reflect different aspects of the complex underlying pathophysiology.

Given the large pathophysiological and phenotypical heterogeneity of DM, obesity, and CV disease, the need for a tailored rather than a “one size fits all” approach in their treatment is increasingly recognized. There is preliminary evidence that gut microbes can serve as predictive biomarkers for the success of weight loss diets [89], highlighting the important role that distinct intestinal microbial signatures could play in the future in the personalized treatment of metabolic diseases. There is a need for well-designed studies that will evaluate the relationship between presepsin levels and individual response to anti-diabetic and anti-obesity medications, as well as other drugs that are used to reduce CV risk, together with lifestyle interventions, including nutritional and exercise plans.

Future research efforts must delve into the evaluation of presepsin in patients with HF, ischemic heart disease, and obesity and prospectively investigate its association with clinically significant outcomes. Another topic of interest is the effects of antidiabetic agents on presepsin levels, with a special focus on drugs that possess anti-inflammatory properties and also act through the gut, such as GLP-1 RAs and dual SGLT 1 and 2 inhibitors, such as canagliflozin and sotagliflozin [90,91]. Our group is actively involved in research in this field, and we hope to soon have convincing answers to interesting questions about the emerging role of presepsin in the management of DM and other cardiometabolic diseases.

## Figures and Tables

**Table 1:** An overview of key studies that investigated presepsin in infection-free subjects with cardiometabolic disorders.

First Author, Year [Ref.]	Study Design	Study Population	Key Findings
Caglar, 2017 [84]	Cross-sectional	48 patients with STEMI and 50 controls	Presepsin levels were higher in STEMI patients compared with controls
Nishimura, 2017 [81]	Prospective	506 patients hospitalized with worsening HF	Presepsin was an independent predictor of 6-month mortality
Biyik, 2018 [74]	Cross-sectional	48 patients with well-controlled HT (22% with DM)/49 normotensive controls (19.6% with DM)	Presepsin levels were lower in patients with HT than controls and positively correlated with hsCRP
Nishimura, 2018 [85]	Prospective	239 outpatients with CKD	The combined assessment of presepsin and BNP predicted CV events
Qi, 2019 [77]	Retrospective	165 out-of-hospital cardiac arrest patients (22.4% with DM)/100 healthy controls (8% with DM)	Presepsin levels were higher in patients than controls. Low presepsin concentrations were an independent prognostic factor for survival and favorable neurological outcomes
Handke, 2019 [79]	Prospective	40 patients with elevated CV risk undergoing non-cardiac surgery	Preoperative presepsin levels were associated with the risk of perioperative MACCEs
Ishii, 2020 [82]	Prospective	1636 patients treated at medical CICUs	Presepsin levels on admission were an independent predictor of mortality
Kouroupis, 2024 [65]	Cross-sectional	10 patients with uncontrolled T1DM/10 patients with well-controlled T1DM/23 patients with uncontrolled T2DM/19 patients with well-controlled T2DM/13 normoglycemic controls	Presepsin was lower in the well-controlled T2DM group compared to the well-controlled T1DM group. Presepsin levels were associated with the duration of DM
Zografou, 2024 [72]	Cross-sectional	10 patients with uncontrolled T1DM/10 patients with well-controlled T1DM, and 12 participants used CGM	No differences in presepsin levels between the well-controlled and uncontrolled groups. Presepsin was negatively correlated with TIR and positively correlated with GMI and GRI
Koufakis, 2025 [71]	Cross-sectional	27 individuals with obesity, 34 with overweight and 20 lean controls, all free of DM	Presepsin levels were higher in the obesity group compared to the overweight and control groups

Abbreviations: DM: diabetes mellitus; HT: hypertension; hsCRP: high sensitivity C reactive protein; T1DM: type 1 diabetes mellitus; T2DM: type 2 diabetes mellitus; CGM: continuous glucose monitoring; TIR: time in range; GMI: glucose management indicator; GRI: glycemia risk index; CV: cardiovascular; MACCEs: major adverse cardiovascular and cerebrovascular events; HF: heart failure; CICUs: cardiac intensive care units; STEMI: ST elevation myocardial infarction; CKD: chronic kidney disease; BNP: B-type natriuretic peptide.

## Data Availability

No new data were created or analyzed in this study. Data sharing is not applicable to this article.
